# Optimization of Monolayer MoS_2_ with Prescribed Mechanical Properties

**DOI:** 10.3390/ma15082812

**Published:** 2022-04-12

**Authors:** Wacław Kuś, Mohammed Javeed Akhter, Tadeusz Burczyński

**Affiliations:** 1 Department of Computational Mechanics and Engineering, Silesian University of Technology, 44-100 Gliwice, Poland; 2 Institute of Fundamental Technological Research, Polish Academy of Sciences, 02-106 Warsaw, Poland; mjakhter@ippt.pan.pl (M.J.A.); tburczynski@ippt.pan.pl (T.B.)

**Keywords:** optimization, MoS_2_, nanostructure, mechanical properties

## Abstract

Various technological challenges are essentially material problems in our times. New functional and functional graded nanomaterials are constructed of components with predefined properties. The design of nanostructures with predefined mechanical properties was considered in this paper. This study applies the evolutionary algorithm (EA) to the optimization problem in the design of nanomaterials. The optimal design combined EA with molecular dynamics to identify the size of the void for the prescribed elastic properties in monolayer 2D MoS_2_ nanostructures. The numerical results show that the proposed EA and the use of optimization method allowed accurately obtaining nanostructures with predefined mechanical material properties by introducing elliptical voids in the 2D MoS_2_ nanosheets.

## 1. Introduction

In the ever-growing nanotechnology field, materials with unique properties have continuously been presented to meet the growing demand for practical industries and engineering. Therefore, it becomes vital to investigate the material properties of these nanomaterials to provide information to understand and then design nanostructures. Studies on the numerous properties of monolayer 2D molybdenum disulfide MoS_2_ nanomaterial have attracted many researchers in this field. Transition metal dichalcogenide (TMD) flat MoS_2_ is a triple layer of molybdenum and sulfur atoms arranged in a hexagonal crystal lattice. It has excellent mechanical [1] electrical and chemical properties [2,3]. Consequently, MoS_2_ has been the focus of substantial research in recent years, ensuring that the material can be used in a wide range of emerging technologies and future applications, including, for example, nanoporous MoS_2_ membranes for efficient reverse osmosis desalination and gas separation, membrane separation, desalination of water [4], DNA sequencing [5], and power generation [6,7]. Therefore, the properties and behavior of monolayer MoS_2_ must be understood and accurately predicted under various conditions to introduce MoS_2_ to novel applications. However, fabricated MoS_2_ sheets typically contain a variety of defects, including nanopores [8,9]. Thus, microstructural voids can profoundly impact the properties of MoS_2_, which helps design the nanomaterial, ultimately influencing the performance of MoS_2_-based devices. Likewise, during the growth and processing of MoS_2_, different topological defects (such as vacancies, inclusions, dislocation, and grain boundaries) and other sizable defects (such as nano-holes and nano-cracks) are inevitable, which can compromise the expected performance in the preparation and handling of MoS_2_-based nanodevices [10]. In addition, such defects can also occur due to the conditions in which MoS_2_-based devices are used [11]. Functional materials in nanosystems are often based on well-known materials but with tuned material properties. The 2D MoS_2_ sheet can be functionalized by introducing voids of appropriate sizes. The sizes of the voids determine the mechanical properties of the material. In recent years, researchers have carried out investigations on the mechanical performance of monolayer MoS_2_ related to aspects such as defects, inclusions, strength, damage, debonding, and failure that greatly influence its properties [1,12].

This work aims to study the identification problem in order to find the parameters that describe the shape of the void as a function of the properties of the prescribed material. This approach can be used in future applications where functional or functional graded materials or nanomaterials can be designed. Materials with a priori prescribed material properties can be created by the introduction of voids. The method presented in the paper is based on two components: a developed optimization algorithm and a direct problem solver. The nanostructures were simulated using the molecular dynamics (MD) method. Monolayer MoS_2_ with various mechanical properties can be computed by molecular statistics or MD methods with a set of numerical tests comprising uniaxial tension, compression, and shear [1,13,14,15]. The well-known MD code LAMMPS [16] was used in this study for direct problem solving. In recent years, inverse methods have been widely applied to predict the properties of structures and materials. These methods allow solving problems regarding parameters using optimization techniques and a set of direct problem solutions. Inverse methods were used for mechanical and thermomechanical problems in which the properties of materials and their structures were searched [17]. Inverse problems can be solved using direct problem formulations computed using numerical methods such as the finite element method (FEM), boundary element method (BEM), and MD. The objective function of optimization algorithms in inverse methods is, in most cases, multimodal; thus, global optimization techniques are often used during the problem-solving process. Applications of new approaches of evolutionary algorithms coupled with BEM computation in optimization and identification for cracked structures and internal void defects under thermomechanical and dynamical loading were shown in [17,18,19]. Sigmund [20] used the inverse homogenization method to tune the elastic properties of the material for periodic truss, frame, and continuum structures, as well as design microstructures with prescribed elastic properties and negative Poisson’s ratios. An in-house implementation of EA was used to search for new stable molecular graphene-like 2D materials [21,22,23].

In our earlier work [1], we computed the mechanical properties, i.e., independent elastic properties, for monolayer MoS_2_ with single and multiple random defects. The computational results from our previous work showed the significant influence of defects on the elastic material properties of MoS_2_ nanosheets. In this work, we aimed to identify the void size for the prescribed elastic properties defined by the user. We intuitively define the prescribed elastic properties, assuming that the sheet contains a void, which should be lower than the material without a void. Thus, we use the tools discussed earlier in the implementation of EA coupled with LAMMPS. As a result, we obtained the size of the void for the prescribed elastic properties.

The remainder of this work is arranged as follows: Section 2 presents the evolutionary optimization and the evaluation of the objective function. Section 3 details the molecular dynamics modeling of 2D MoS_2_ with voids and the evolutionary identification of voids with prescribed properties by minimizing the objective function. Numerical identification examples proving the ability of this method in solving the intended optimization problem iteratively are also provided.

## 2. Materials and Methods

### 2.1. Optimization Problem Formulation

**Figure 1:**
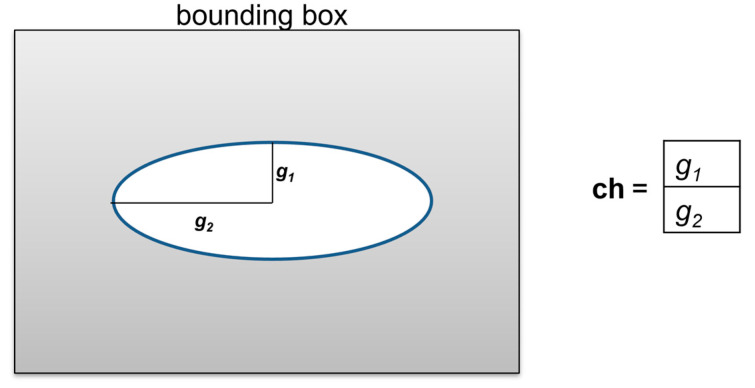
The nanostructure with an elliptical void described by two design variables.

The goal of the optimization problem is to design in an automatic way MoS_2_ structures with predefined material properties. The mechanical stiffness is taken into account in this paper; however, the optimization problem can also be solved for thermal, optical, or other properties of the microstructure. The objective function depends on the prescribed material properties and the actual properties computed for each design of the microstructure. The design vector **ch** may define the size, shape, and topology of the microstructure. The MoS_2_ structure considered in this paper was modified by introducing voids with the properties described using design variables $\;g_{i}$. The optimization problem can be formulated as

(1) $$\{\begin{array}{c} find\;\;\;\;\;\;\;\;\;\;\;\;\;\;\mathrm{ch}=\left(g_{1},g_{2},\ldots \ldots g_{N}\right)\\ minimize\;\;\;\;\;\;\;f\left(\mathrm{ch}\right)=\|P-P_{ref}\|\\ s.t\;\;\;\;\;\;\;g_{iL}\;\leq g_{i}\leq \;g_{iU} \end{array},$$

where $g_{iL}$ and $g_{iU}$ are the lower and upper constraints of design variables. Tensor or vector-containing tensor element **P** denotes the nanostructure properties obtained for design vector **ch**, and $P_{ref}$ describes the prescribed reference properties of the nanostructure. The goal of minimizing the difference between current and reference material properties is set as a function of design variables leading to an objective function equal to zero (i.e., identical reference and obtained properties). Optimization with a small difference between the reference and obtained properties is also acceptable. The paper is devoted to the optimization of the nanostructure taking into account mechanical properties (in our case, P and $P_{ref}$**)** depending on the stiffness of the nanostructure with introduced voids. The stress–strain relationship for small strains can be expressed with Voigh notation as follows:

(2) $$\sigma_{ij}=P_{ij}\varepsilon_{ij},$$

where $\varepsilon_{ij}$ denotes the strain components, and $P_{ij}$ denotes the elastic constants to be used during objective function evaluation. The shape of the nanostructure is modified according to **ch** by introducing an elliptic void, as shown in Figure 1.

**Figure 2:**
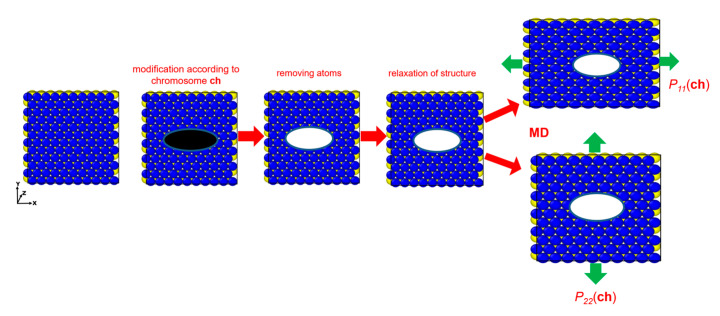
Determination of nanostructure stiffness in two directions. An ellipse is introduced into the pristine nanostructure by removing atoms in black area. Next, the structure is relaxed, and two analyses of microstructure stretching are performed in two different directions. Then, the stiffness is computed on the basis of the MD results.

The 2D infinite nanostructure was modeled using periodic boundary conditions, and the maximum size of the void was defined by the unit cell size used in simulations. The mechanical properties (stiffness in two directions) were computed using the MD approach. The algorithm for determining the stiffness of the nanostructure is shown in Figure 2.

First, the void was introduced into the pristine 2D nanostructure. Next, relaxation was performed, and the stress–strain curve was obtained in two directions during uniaxial tensile load. In general, other loads can be applied to obtain, for example, shear stresses or thermal and optical properties of the nanostructure.

**Figure 3:**
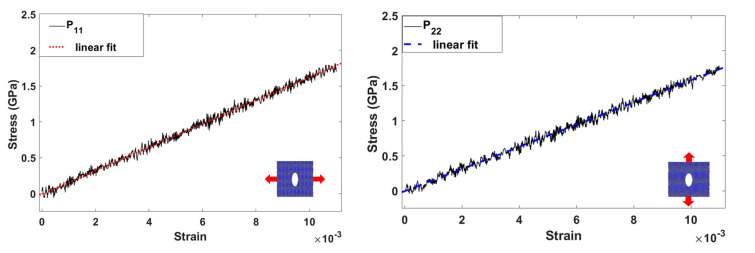
Examples of stress-strain relationship during uniaxial tension in two directions.

This paper contains examples of optimization based on the computation of an objective function using MD results for stiffness computed in two directions for monolayer MoS_2_. The atomic model consisted of about 10,000 atoms in the nanosheet for a domain size of 175 Å^2^. The Stillinger–Weber (SW) [24,25] potential was applied to describe interatomic interactions between atoms. Before tensile deformation, the model was relaxed at 300 K and 0 bar pressure through an isothermal–isobaric ensemble (NPT) for 30 ps (picoseconds). All MoS_2_ tensile deformations were carried out at a constant temperature of 300 K. The Nose–Hoover thermostat was used to maintain the temperature of the simulation system. The position and velocity of all atoms were updated by the Verlet integration algorithm. The LAMMPS software package and the Open Visualization Tool (OVITO) [26] were used for molecular dynamics simulation, visualization, and output data analysis. Uniaxial tensile deformation at a constant strain rate of 0.0001 ps^−1^ was applied to estimate the stiffness of the structure. The stress tensor components [27] were calculated as follows:

(3) $$\sigma_{ab}=\frac{1}{V}\left[\frac{1}{2}\overset{N^{*}}{\underset{i}{\sum }}\overset{N}{\underset{j\left(\neq 1\right)}{\sum }}f_{ij}^{a}r_{ij}^{b}+m_{i}u_{i}^{a}u_{i}^{b}\right],$$

where *a* and *b* denote the Cartesian components, *f_ij_* is the force acting on atom *i* due to another atom *j*, *V*, *m_i_*, and *u_i_* are the volume, mass, and velocity of atom *i*, and *N* is the number of atoms. Figure 3 shows examples of two simulation results with a stiffness of 163 GPa and 158 GPa.

The objective function value could be computed for such a case as the sum of the absolute difference between computed stiffness and prescribed material properties.

### 2.2. Evolutionary Optimization

Optimization can be defined as selecting the best choice (according to some criteria) from a set of available variants. There are mainly two groups of optimization methods. The first group includes local optimization methods, mostly leading to the local optimum, where the results depend on the starting vector values. The second group includes global optimization techniques, mostly based on heuristics. Heuristic methods are best suited for multimodal functions, where most algorithms fail to determine the global optimum. Many optimization techniques are based on biologically inspired methods such as natural selection, learning procedures, and probabilistic rules. Evolutionary algorithms [28] and particle swarm optimization (PSO) [29] are examples of these techniques. These techniques can solve multimodal optimization problems in mathematics and engineering. It is impossible to identify the best optimization techniques among those mentioned above, since each algorithm has its strengths and weaknesses, and the performance depends on the optimization problems, constraints, and algorithms parameters. The evolutionary algorithm (EA) searches the space of possible solutions on the basis of mechanisms taken from the evolution of species.

**Figure 4:**
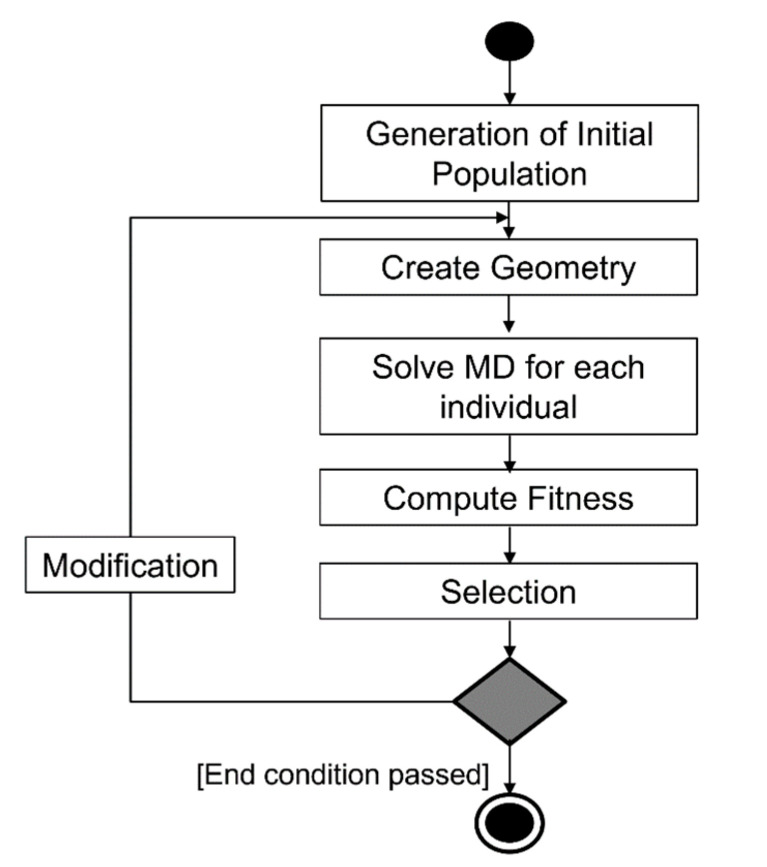
Evolutionary algorithm flowchart.

The flowchart of our implementation of EA used in this work is shown in Figure 4. The algorithm starts by generating an initial population of individuals generated in a random or ordered way according to the requirements of the problem. The individual, containing chromosomes, represents a single solution. Usually, in applications of EA, individuals contain only one chromosome **ch**, with vectors of genes representing design variables *g_i_*. Genes may contain coded design variables; however, in our approach, we used floating point genes. Hence, additional coding was not needed.

For each individual, MD simulations and individual fitness functions were calculated in the next step of the algorithm on the basis of the atomic structure defined by genes. The selection procedure was performed to choose individuals for the next iteration considering their fitness values. The probability of survival of an individual depends on the value of the fitness function. An individual with good fitness has a better chance of survival during the selection process. The ranking selection is performed in a few steps. First, individuals are classified according to the values of the fitness function; then, a rank value is assigned to each individual. This depends on the individual’s number and the rank function. The best individuals obtain the highest rank value; the worst individuals obtain the lowest ones. In the final step, individuals for the offspring generation are drawn, but the probability of drawing particular individuals is closely related to their rank value. The process is repeated iteratively until the termination condition is satisfied. The termination condition may be formulated as a maximum number of iterations. In cases when the stop condition is not fulfilled, the genes are modified using evolutionary operators. The algorithm in this paper used evolutionary operators such as uniform and Gaussian mutation, as well as simple and arithmetic crossover. The uniform and Gaussian mutations modify individuals randomly. Simple and arithmetic crossovers create new chromosomes on the basis of two randomly chosen chromosomes from the population. The modified individuals are introduced into the population.

The objective function (i.e., the fitness function for each chromosome) is computed on the basis of a direct problem solution for each individual. The number of direct problems solved in EA is quite large due to the number of chromosomes and the number of iterations of the algorithms. To minimize the total optimization time (wall time), a parallel approach is crucial, and it was used to compute the numerical examples presented in this paper.

## 3. Results

Nanostructure optimization by introducing a void according to prescribed mechanical properties is illustrated using a few cases in the section. The tests were performed for a few sets of prescribed material properties. The evolutionary algorithm featured two subpopulations, a total number of individuals of 32, tournament selection with a tournament size of 5, uniform selection with a probability of 0.3, Gaussian mutation with a probability of 0.5, simple crossover with a probability of 0.1, and arithmetic crossover with a probability of 0.1. The parameters of the EA were chosen on the basis of previous experience gained during the solving of structural optimization problems [19,21,22,23]. The number of EA iterations was set as 50.

An optimized nanostructure with an approximate size of 170 Å × 170 Å containing an elliptical void was used. The size of the void could range from 1 × 1 up to 50 × 50 Å (radius of the elliptic void). The stop condition was formulated as the maximum number of iterations.

The evaluation of the fitness function for each individual was performed as described in Section 2. The LAMMPS software was used to solve two problems to obtain the stiffness in two directions, and the objective function was calculated on the basis of these results. The computing of the fitness functions in each iteration can be performed in parallel way with very good efficiency when the number of processing units is equal to the number of MD problems (number of individuals times number of mechanical properties; in our case, 64). Additional processing units may also be used for parallelizing each MD simulation. Such an approach can be used to perform computations with a high number of processing units. The results presented in the section were obtained with the use of supercomputers Okeanos and Karolina. Hundreds to thousands of processor cores were used during computations.

**Figure 5:**
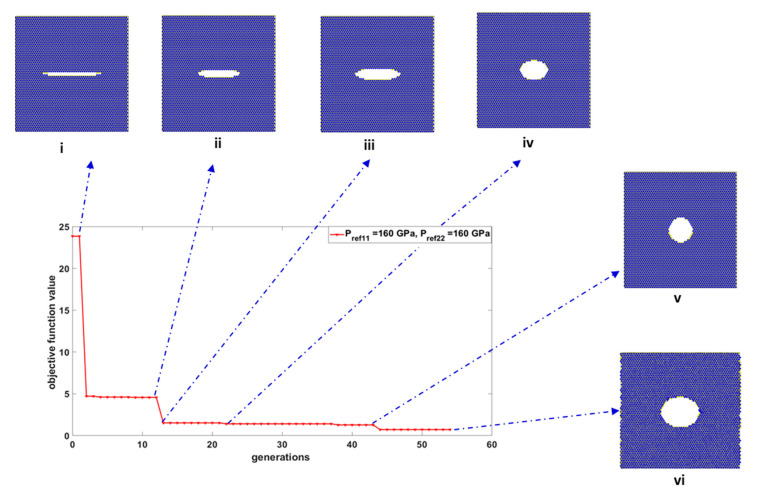
Progress of the convergence of the objective function indicating the evolution of the void during iterative generations for prescribed elastic properties *P*_*ref*11_ = 160 GPa, *P*_*ref*22_ = 160 GPa. The corresponding void dimensions obtained during generations were (i) *g*_1_ = 46.06 Å and *g*_2_ = 2.95 Å, (ii) *g*_1_ = 32.18 Å and *g*_2_ = 6.07 Å, (iii) *g*_1_ = 33.92 Å and *g*_2_ = 8.86 Å, (iv) *g*_1_ = 21.36 Å and *g*_2_ = 14.88 Å, (v) *g*_1_ = 18.41 Å and *g*_2_ = 19.38 Å, and (vi) *g*_1_ = 28.12 Å and *g*_2_ = 23.15 Å.

The changes in the best objective function over few iterations for adequate nanostructures with prescribed material properties *P*_*ref*11_ = *P*_*ref*22_ = 160 GPa are shown in Figure 5.

A total of three distinct void identification analyses were performed for the MoS_2_ nanosheet with different prescribed material properties. The void (elliptical void) was induced at the center of the sheet. Table 1 contains the values of the best obtained solutions for the numerical tests. The obtained material properties (*P*_11_, *P*_22_) for the ellipse radius (*g*_1_, *g*_2_) were not identical to those prescribed (**P***_ref_*), as denoted by the errors *eP*_11_ and *eP*_22_. The obtained properties were very close to the prescribed ones.

**Figure 6:**
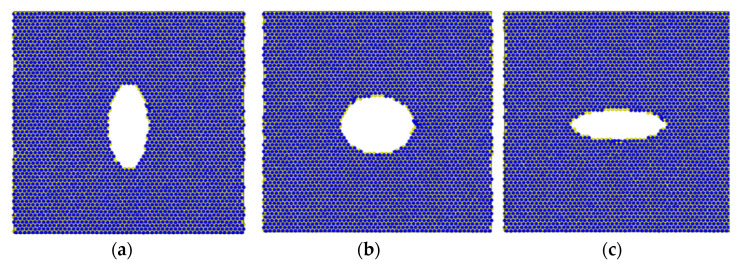
Monolayer MoS_2_ nanosheet with void identified by optimization: (**a**) *P*_*ref*11_ = 180, *P*_*ref*22_ = 150 GPa (*g*_1_ = 15.35 Å and *g*_2_ = 33.44 Å); (**b**) *P*_*ref*11_ = 160, *P*_*ref*22_ = 160 GPa (*g*_1_ = 28.12 Å and *g*_2_ = 23.15 Å); (**c**) *P*_*ref*11_ = 150, *P*_*ref*22_ = 180 GPa (*g*_1_ = 36.35 Å and *g*_2_ = 12.19 Å).

The resulting structures for the above cases are shown in Figure 6. The nanostructure in the first case should have an ellipse with a bigger radius in the *y*-direction; the radii in in the second case should be similar, whereas those in the third case should be similar to the first case when considering the prescribed material properties. The MoS_2_ nanostructure was not symmetric in the *x* (*g*_1_) and *y* (*g*_2_) directions; thus, we did not expect the same ellipse radii for the second case in the *x-* and *y*-directions. The results agreed with the intuitive approach and gave exact values of void size. The method can be used for any prescribed stiffness values; however, it is of course limited by the size of the void and maximum stiffness of the MoS_2_ sheet.

## 4. Conclusions

The appearance of defects in MoS_2_ can weaken its mechanical properties, such as fracture strength and Young’s modulus. However, such defects have potential in novel applications of MoS_2_, such as graded materials or nanosystems. We used the optimization method to tune the material properties for a periodic monolayer MoS_2_ with a void as a defect. The tailoring problem was formulated as an optimization problem to identify the simplest possible nanostructure with constraints given by the prescribed elastic constants. Numerical examples proved that the proposed method could find the void size with prescribed mechanical properties. The present study shows the potential of molecular simulations for 2D nanostructures. It is revealed as an efficient method to design nanostructures with prescribed properties. The method is generally applicable and can be used to take into account the thermal or optical properties of the nanostructure by modifying the components of the objective function and using an adequate direct problem method.

## Figures and Tables

**Table 1:** The prescribed and resulting stiffness, ellipse radius, and error of obtained stiffness.

*Case*	*P*_*ref*11_ (GPa)	*P*_11_ (GPa)	*P*_*ref*22_ (GPa)	*P*_22_ (GPa)	*g*_1_ (Å)	*g*_2_ (Å)	*eP*_11_ (%)	*eP*_22_ (%)
1	150.0	149.2	180.0	179.5	36.35	12.19	0.5	0.3
2	160.0	162.6	160.0	158.0	28.12	23.15	1.6	1.3
3	180.0	179.5	150.0	148.0	15.35	33.44	0.3	1.3
